# Liquid Biopsy in Head and Neck Cancer: Current Evidence and Future Perspective on Squamous Cell, Salivary Gland, Paranasal Sinus and Nasopharyngeal Cancers

**DOI:** 10.3390/cancers14122858

**Published:** 2022-06-09

**Authors:** Santiago Cabezas-Camarero, Pedro Pérez-Segura

**Affiliations:** Medical Oncology Department, Hospital Clínico Universitario San Carlos, Instituto de Investigación Sanitaria San Carlos (IdISSC), 28040 Madrid, Spain; perezsegura09@gmail.com

**Keywords:** liquid biopsy, head and neck cancer, circulating tumor cells, CTC, circulating tumor DNA, ctDNA, human papillomavirus, HPV, Epstein-Barr virus, EBV, extracellular vesicles, microRNAs

## Abstract

**Simple Summary:**

Head and neck cancer is the sixth most common type of solid tumor and harbors a poor prognosis since most patients are diagnosed at an advanced stage. The study of different tumor components in the blood, saliva or other body fluids is called liquid biopsy. The introduction of novel diagnostic tools such as liquid biopsy could aid in achieving earlier diagnoses and more accurate disease monitoring during treatment. In this manuscript, the reader will find an in-depth review of the current evidence and a future perspective on the role of liquid biopsy in head and neck cancer.

**Abstract:**

Head and neck cancer (HNC) is currently the sixth most common solid malignancy, accounting for a 50% five-year mortality rate. In the past decade, substantial improvements in understanding its molecular biology have allowed for a growing development of new biomarkers. Among these, the field of liquid biopsy has seen a sustained growth in HNC, demonstrating the feasibility to detect different liquid biomarkers such as circulating tumor DNA (ctDNA), circulating tumor cells (CTC), extracellular vesicles and microRNAs. Liquid biopsy has been studied in HPV-negative squamous cell carcinoma of the head and neck (SCCHN) but also in other subentities such as HPV-related SCCHN, EBV-positive nasopharyngeal cancer and oncogene-driven salivary gland cancers. However, future studies should be internally and externally validated, and ideally, clinical trials should incorporate the use of liquid biomarkers as endpoints in order to prospectively demonstrate their role in HNC. A thorough review of the current evidence on liquid biopsy in HNC as well as its prospects will be conducted.

## 1. Introduction

Head and neck cancer (HNC) is the sixth most common solid tumor and is the eighth most common cause of mortality [1]. Sixty percent of HNCs present with locally advanced disease. Unfortunately, up to 50% of these patients will relapse within the next three years, in most instances as an incurable disease [2]. In addition, HNC is one of the cancers with the highest impact on quality of life and social functioning due to the anatomic and functional areas involved [3]. Earlier disease diagnosis and identification of relapse would help to improve this poor prognosis and the functional outcomes after therapy. The field of liquid biopsy has been one of the leading research areas in oncology during the past fifteen years. While more broadly developed in breast, lung, colorectal and prostate cancers, notable milestones have been attained in other less common entities such as head and neck cancer (HNC). Being the sixth most common solid tumor and the eighth most common cause of cancer death worldwide, improvements in understanding its biology and the refinement of molecular diagnostics have paved the way for the introduction of liquid biopsy in HNC. A growing number of studies using different technologies, such as circulating tumor cells (CTCs), circulating tumor DNA (ctDNA), micro-RNAs (miRNAs) and extracellular vesicles (EVs), have demonstrated the feasibility of applying liquid biopsy as a prognostic and/or predictive tool in patients with HNC [4]. However, the lack of internally and externally validated studies, as well as the scarcity of prospective liquid biomarker trials in HNC, limits our understanding of liquid biopsy in this disease and prevents its widespread incorporation into clinical practice [5]. On the other hand, although HNC’s complex biology and lack of frequent hotspot mutations limit its development as a predictive tool, a growing number of studies have demonstrated the predictive value of CTCs and/or ctDNA in HPV-unrelated squamous cell cancer of the head and neck (SCCHN) as well as in certain sub-entities such as human papillomavirus (HPV)-related oropharyngeal cancer (OPC), Epstein-Barr Virus (EBV) nasopharyngeal cancer (NPC) and certain salivary gland carcinomas [6,7,8].

The present review will summarize the current state-of-the-art of liquid biopsy in HNC as well as its future perspectives, and describe both the available evidence for using different liquid biopsy modalities and the upcoming advances in the field [9,10,11,12,13,14].

## 2. Current Evidence

### 2.1. Circulating Tumor Cells (CTC)

#### 2.1.1. CTC in Squamous Cell Carcinoma of the Head and Neck

CTC investigations in patients with HNC have used different methods from one another within small-sample sized studies. Besides, many of them have been limited to CTC counting to use them as a prognostic tool, with only a few studies having sought their molecular characterization.

Most studies have used detection methods based on immunoaffinity with positive or negative enrichment depending on each case. However, a growing number of studies have also been reported with CTC detection methods based on size or other physicochemical characteristics [15,16,17,18,19,20,21,22,23,24,25,26,27,28,29,30,31,32,33,34,35,36,37,38,39,40,41,42,43,44,45].

Hristozova et al. [26], using flow cytometry, detected the presence of CTCs in 18/42 (43%) patients with unresectable LA SCCHN, finding that the detection of CTCs correlated with the presence of locoregional lymph node metastases. Gröbe et al. [34], using the CellSearch system, detected CTCs in 12.5% of 110 patients with oral cavity cancer who had undergone R0 surgery, finding that the presence of CTCs was associated with a shorter PFS, a higher T and the presence of locoregional metastases. Tinhofer et al. [27], using a method based on the detection of CTCs through the amplification of tumor antigens, detected CTCs prior to the start of adjuvant treatment in 29% of 144 patients with resected LA SCCHN. The presence of CTCs was higher in tonsil or tongue base tumors, and although there was a non-significant trend towards better disease-free survival (DFS) in oropharyngeal carcinomas, there was a significant association between non-oropharyngeal carcinomas and worse DFS and OS. Finally, Grisanti et al. [28], using the CellSearch ™ system, identified CTCs in 26% of 53 patients with R/M SCCHN, finding that the cut-off point of ≥1 CTCs was associated with a worse PFS and OS, achieving a significantly higher disease control rate in patients without CTCs than with CTCs (45% vs. 8%, *p* = 0.03). In the prospective CIRCUTEC study, 65 patients with R/M SCCHN were included, with blood samples being taken at baseline and on the 7th and 21st days after starting the first CT cycle with the EXTREME regimen (cisplatin-5FU-cetuximab three weeks). Three CTC detection systems were used: CellSearch, EPISPOT and flow cytometry (FCM). EPISPOT, CellSearch and FCM detected CTC in 69%, 21%, and 11% of the cases, respectively. PFS was significantly shorter in patients with stable or increasing CTC numbers between baseline and 7-day determination with EPISPOT (3.9 vs. 6.2 m; 95% CI 5–6.9, *p* = 0.0103), and the same occurred in patients with CTC ≥ 1 with EPISPOT or with CellSearch (*p* = 0.03), with EPISPOT or FCM (*p* = 0.048), and CellSearch or FCM on day 7 (*p* = 0.0005). This study therefore suggests that CTCs can be used to monitor early response to chemotherapy in R/M SCCHN [31].

In a prospective cohort of 113 patients with LA SCCHN, the CellSearch system was used to determine CTCs at baseline, after 2 cycles of induction CT and at the end of treatment with chemoradiotherapy (CRT). PD-L1 overexpression in CTCs was detected in 25.5%, 23.5% and 22.2% of the patients at each of the moments described, respectively. Patients with PD-L1 overexpression CTCs at the end of CRT had worse PFS (*p* < 0.001) and OS (*p* < 0.001), and it was an independent prognostic factor in multivariate analysis. The authors proposed that PDL1 + CTCs could be used to identify a population of patients that could benefit from adjuvant treatment with checkpoint inhibitors directed against the PD1-PDL1 axis [35]. Along the same lines, Chikamatsu et al. [32] isolated CTCs from 30 patients with R/M SCCHN by negative depletion of leukocyte cells using magnetic particles and the identification of CTCs by mRNA expression of various epithelial markers (CK19, EpCAM, EGFR and c-MET). In addition, the expression in CTCs of PD-L1, PD-L2 and CD47 was analyzed. Positive CTCs were detected for the expression of at least one epithelial marker in 24 cases (80%), and of these, the expression of CD47, PD-L1 and PD-L2 occurred in 79.2%, 83.3% and 70.8%, respectively. There was no correlation between the expression of PD-L1 in the tumor and in the CTCs. In a patient with metastatic squamous cell carcinoma of the larynx, Kulasinghe et al. [33] demonstrated the detection of CTC “clusters” using a spiral microfluidic system, which after staining with specific antibodies were shown to express PD-L1.

This same group compared the CellSearch system with two other non-immunoaffinity-based detection systems, the ScreenCell system and the RosetteSep in patients with SCCHN. CTC were detected in 18.6% (8/43), 46.4% (13/28) and 64% (16/25) of the cases, respectively, and they demonstrated that the two non-IA-based methods detected CTC in a greater number than CellSearch [36].

Morgan et al. [43] used a novel spectroscopy technology which has been used in the development of space research and is and known as Surface-Enhanced Raman Scattering (SERS) nanotechnology. EGF (epidermal growth factor) was used as the capture antigen. First, low-density CTCs and leukocytes were isolated by density gradient centrifugation and were then incubated with SERS-EGF nanoparticles, before being illuminated with a 785 nm laser. The number of CTCs could be determined as a function of the SERS signal intensity. With this system, the number of CTCs detected in 82 patients with LA SCCHN was considerably higher than with other technologies, establishing 675 CTCs as the best cut-off point to predict distant metastasis-free survival (DMFS) at 1, 2 and 5 years.

In a meta-analysis published in 2017, which included a total of 17 studies with CTCs in patients with SCCHN, it was concluded that the presence of CTCs is significantly statistically associated with poor OS (HR 2.8, 95% CI 1.34–5.86), SLE (HR 3.86, 95% CI 2.03–7.36) and PFS (HR 3.31, 95% CI 1.71–6.42). In addition, patients with CTCs tend to relapse more, and present locoregional lymph node metastases and a more advanced T category [44].

The PREDICT-HN (NCT03491176) is an ongoing study in patients with SCCHN who receive treatment with CRT with radical intention and who undergo weekly MRI imaging studies and weekly blood draws for the study of CTCs by the CellSearch and ctDNA system. The main objective is to evaluate the value of tumor response kinetics and CTCs to predict response to treatment [45]. Table 1 summarizes the most relevant studies with CTC in patients with SCCHN.

#### 2.1.2. CTC in EBV+ Nasopharyngeal Cancer

Some groups have studied the role of CTCs in patients with NPC [46,47,48,49,50,51,52,53,54,55,56]. Table 2 summarizes the most relevant studies with CTC in patients with NPC.

Zhang et al. [46], in a cohort of 50 patients with NPC stage II-IV, used the SE-iFISH technology for CTC detection before and after chemotherapy; they achieved a 92% CTC detection rate and demonstrated a higher CTC detection in more advanced stages. A decreased CTC count and chromosome 8 aneuploidy in CTC after chemotherapy was associated with tumor response. The same group used ISET technology in 33 stage II-IV NPC patients to demonstrate a 66.7% detection rate with a good correlation with EBV viral load [46]. Si et al. [47], using the CanPatrol™ system in 81 stage I-IV NPC patients, demonstrated a detection rate of 96.6% and a higher CTC count in N+ and M1 disease.

You et al. [48] compared CTC versus EBV-ctDNA pre- and post-chemotherapy in 148 R/M NPC patients and in 122 LA NPC patients, demonstrating that the prognostic role of both CTC and ctDNA improved when both are used together and when combined with imaging tests. 

Li et al. [49] studied CTC detection and COX-2 expression in CTC in 131 LA NPC patients, demonstrating a 66.4% and 46.1% COX2+ CTC detection rate before and after chemoradiotherapy, respectively. In the latter case, this was associated with a worse prognosis.

Ou et al. [53], using the CellSearch system in 370 patients with stage III-IV NPC, detected CTC in 27% and 20% of non-metastatic and metastatic patients, respectively, demonstrating a worse prognosis with a higher CTC count. Combining CTC and EBV-ctDNA improved their prognostic value. 

Sun et al. [54] compared EBV-ctDNA versus CTC detection with the CellSpoter Analyzer technology in 144 non-metastatic and 136 metastatic NPC patients, and found CTCs in 9.3% and 12.5%, respectively.

Several other smaller studies have also demonstrated the prognostic value of CTC in patients with NPC, and these are summarized in Table 2 [46,47,48,49,50,51,52,53,54,55,56].

#### 2.1.3. CTC in Salivary Gland Cancer

Very few studies have been published regarding the use of liquid biopsy in salivary gland cancers, despite their frequently redundant hotspot alterations in certain entities such as salivary duct carcinoma (SDC), mucoepidermoid carcinoma (MEC), NTRK-driven mammary analog secretory carcinoma (MASC) or adenoid cystic carcinoma (ACC). Common alterations include HER2 amplification, androgen receptor (AR) overexpression, MYC amplification, PIK3CA mutations and NTRK fusions [57,58,59,60]. In a pilot study of 8 patients with ACC using a spiral microfluidics-based isolation method and detection by immunofluorescence, 3 patients (32.5%) were positive for CTC, each patient with either metastatic or recurrent disease [61]. In a case reported by Cappeletti et al. [62], in a patient with metastatic SDC, using the CTC-enrichment method Parsortix followed by single-cell identification and recovery with the DEPArray technology demonstrated the AR splicing variant 7 (Arv7) in CTC and predicted the development of resistance to abiraterone therapy 6 months in advance.

#### 2.1.4. CTC in Paranasal Sinus Cancer

Paranasal sinus cancers encompass a diverse family of entities ranging from squamous cell carcinomas to adenocarcinomas and undifferentiated carcinomas. Among adenocarcinomas, two main reference types exist: intestinal-type adenocarcinomas (ITAC) and non-ITAC type. Unfortunately, there is a major lack of evidence on the use of liquid biopsy in paranasal sinus cancer. Our group published the case of a patient with recurrent ITAC in whom liquid biopsy using the IsoFlux EPCAM-based microfluidic technology detected 26 CTC. Genomic analysis of CTC DNA using castPCR revealed a mutation in KRAS G12A that was retrospectively also detected in the primary tumor using BEAMing technology [63].

### 2.2. Circulating Tumor DNA (ctDNA)

#### 2.2.1. ctDNA in HPV-Unrelated Squamous Cell Carcinoma of the Head and Neck

Lin et al. [64] studied ctDNA in 121 patients with oral cavity SCCHN and in 50 healthy controls. The level of ctDNA was significantly higher in patients with SCCHN than in controls, and it was correlated with tumor size, the presence of regional lymph node metastases and with more advanced stages. Perdomo et al. [65] studied mutations in ctDNA in 5 genes (TP53, NOTCH1, CDKN2A, CASP8, PTEN) using NGS, in 36 patients with stages II-IV SCCHN. In 42% of the cases, mutations were detected in the ctDNA, and 67% of these corresponded to stage I and II patients. A total of 18 matching mutations were detected between plasma and the primary tumor. In another series of 37 patients with stages III and IV SCCHN, complete TP53 sequencing was performed in tumor, plasma and oral rinse samples, as well as in 49 healthy controls. Thirty-six TP53 mutations were detected in tumor, three in plasma, and twenty-six in oral rinses. In 67%, at least 1 mutation in TP53 was detected in the tumor. The concordance of TP53 mutations between tumor, mouthwashes (11%), and plasma (2.7%) was low. The proportion of positive cases in oral rinses was higher in tumors of the oral cavity or oropharynx than in tumors of the larynx. Fostira et al. [66], using the SafeSEQ NGS technology, found a 51% and 93% tumor-plasma concordance in LA and R/M SCCHN, respectively. 

Another very promising application of ctDNA is the detection of minimal residual disease (MRD) after curative-intent therapy. Flach et al. [67], in a single-center prospective cohort study used whole exome sequencing of FFPE samples and compared the mutational profile with that acquired from pre- and post-surgical plasma samples analyzed through deep sequencing using RaDaR^TM^ technology. Among 17 patients, ctDNA was detected in all of the patients prior to surgery, and only in very low levels in post-surgery samples. In all cases with clinical relapse, ctDNA was positive between 108 and 253 days post-surgery. This approach demonstrates the feasibility of using personalized ctDNA assays for disease detection prior to clinical relapse. 

Blood tumor mutational burden (bTMB) was analyzed in patients from the EAGLE trial, a second-line trial comparing standard chemotherapy versus the anti-PDL1 durvalumab alone or versus durvalumab plus the anti-CTLA4 tremelimumab. Although the study did not achieve its primary endpoint for a longer OS for any of the immunotherapy arms, in a subgroup analysis, a high-bTMB (≥16 muts/mb) predicted a better OS and PFS for patients treated with immunotherapy compared to chemotherapy. In addition, ctDNA positive for mutations in KMT2D or ATM predicted a better OS for patients treated with durvalumab plus tremelimumab versus standard chemotherapy [68]. 

Porter et al. [69], in a study of 60 patients with head and neck cancer using the Guardant360 NGS technology, detected ctDNA alterations in plasma in 66% of patients with SCCHN and in 50% of patients with salivary gland cancer (SGC). TP53 (68%), PIK3CA (34%), NOTCH1 (20%) and ARID1A (15%) were the most common alterations detected in ctDNA. These results were concondant with tumor NGS, although 73% had blood alterations not identified in tissue. Wilson et al. [70], in a study of 75 patients with R/M SCCHN, actinonable ctDNA alterations were detected in 65% of the patients, although tumor-ctDNA concordance was only 13%. ctDNA detection was associated with the extent of disease and with a worse OS.

These and other smaller studies are summarized in Table 3 [64,65,66,67,68,69,70,71,72].

In a systematic review of 16 studies from 4 different countries including 1156 patients and 601 controls, ctDNA methylation was significantly increased in patients with SCCHN. The most frequently studied gene mutations were those in TP53 and the most frequently studied gene methylations were those in CDKN2A, DAPK1, RASSF1 and P15 [73]. Similarly, in another systematic review of 10 studies including 390 samples from patients with SCCHN and 79 control samples, the most studied mutation was TP53 [74].

#### 2.2.2. ctDNA in HPV-Related Squamous Cell Carcinoma of the Head and Neck

There are several recent studies that demonstrate the usefulness of the ctDNA study of specific sequences of the human papillomavirus (HPV). Most studies have been carried out in non-advanced disease, either localized or locally advanced, with detection rates of ctDNA in plasma ranging from 65% to 96%, and also with high specificities, generally between 89% and 100% [6,75,76,77,78,79,80].

Damerla et al. [75], using digital PCR in 97 patients with HPV(+) localized OPC, detected HPV-ctDNA in plasma in 95.6%. Chera et al. [76], using digital PCR in 103 patients with HPV(+) localized OPC, detected HPV-ctDNA in plasma in 89% of the patients. Interestingly, 35% of the patients with <95% clearance of HPV-ctDNA after chemoradiotherapy relapsed, while there were no relapses in those with ≥95% clearance.

Cao et al. [78], in 40 patients with HPV-related localized OPC, demonstrated a 65% plasma ctDNA detection rate. Plasma HPV-ctDNA progressively increased in the 3 patients with metastatic relapse. Dahlstrom et al. [77], in 262 HPV-related localized OPC, showed a 60.5% HPV-ctDNA detection rate and 67% specificity using RT-PCR in plasma. Baseline HPV-ctDNA was associated with the nodal and global stage. Ahn et al. [79] demonstrated in 52 patients with HPV(+) OPC that the detection of HPV-ctDNA in plasma or in saliva after concomitant CRT was associated with a high positive predictive value; those ctDNA-HPV+ patients in plasma or saliva had a relapse-free survival (RFS) and overall survival (OS) significantly shorter than the ctDNA-negative patients in saliva and plasma.

Mazurek et al. [80], in a large cohort of HPV-related OPC, demonstrated a 14% HPV-ctDNA detection rate using RT-PCR in plasma, with a higher plasma ctDNA quantity in HPV-related compared to HPV-unrelated cancers. 

In another study conducted in 21 patients with HPV(+) and HPV(−) SCCHN, HPV DNA sequences and frequent somatic mutations in head and neck cancer were studied in plasma and saliva [6]. The saliva study detected alterations in ctDNA in 100% of oral cavity cancers (OCC), 47% of OPC, 70% of laryngeal cancers and 67% of hypopharyngeal cancers, while the plasma detection rates at these subsites were 80%, 91%, 86% and 100%, respectively. However, when plasma and saliva detection were combined, the identification of molecular alterations in ctDNA occurred in 100% of OCC, 91% of oropharyngeal cancers and in 100% of cancers of the larynx and hypopharynx.

Siravegna et al. [81], in a prospective observational study of 70 patients with SCCHN and 70 healthy controls, analyzed plasma ctDNA with ddPCR for HPV genotypes 16, 18, 33, 35 and 45. Sensitivity and specificity of ctDNA was 98.4% and 98.6%, respectively, demonstrating a higher diagnostic accuracy than the standard physical examination and imaging. Costs of HPV ctDNA detection were 36–38% less and ctDNA diagnosis occurred a median of 26 days earlier than with the standard clinical workup. The same group, in another study of 33 patients with resected HPV+ OPC, found that in patients without pathologic risk factors for recurrence, HPV-ctDNA rapidly decreased at postoperative day (POD) 1. However, in patients with risk factors for macroscopic disease, HPV-ctDNA was overtly elevated at POD 1. This elevation was maintained until the start of adjuvant treatment. Higher levels of HPV-ctDNA were detected with extranodal extension > 1 mm and with an increasing number of affected lymph nodes. Therefore, HPV-ctDNA could aid in individualizing adjuvant treatment after surgery in HPV+ OPC [82].

Akashi et al. [83], in a study of 25 patients with HPV+ OPC, detected HPV-specific ctDNA in plasma in 14 (56%) patients at baseline, and found that this was negative after therapy in all patients. In 2 patients, HPV-specific ctDNA was detected at the time of recurrence (Table 4).

#### 2.2.3. ctDNA in EBV+ Nasopharyngeal Cancer (NPC)

The study of ctDNA in the plasma of EBV+ NPC began nearly 2 decades ago. There are, therefore, several authors who have demonstrated the feasibility of studying the ctDNA of specific regions of EBV in the plasma of patients with NPC, both as a prognostic tool that allows refining the TNM staging system and as a variable that allows the identification of patients who would benefit from more intensive treatment with induction chemotherapy followed by radical CRT instead of radical CRT alone [84,85,86,87,88,89,90,91]. It has even been used in the screening of NPC in an endemic population. In a study carried out in Hong-Kong, 20,349 people were screened, 309 of whom were identified as having detectable levels of EBV-ctDNA in their plasma, initiating a screening program directed by nasofibroscopy and magnetic resonance imaging (MRI) which allowed 34 patients to be diagnosed with NPC, most of whom were in the early stages (stages I and II AJCC 7th Ed.). The survival rate achieved compared favorably with the overall number of patients diagnosed in 2013 in the same area, the majority in stages III and IV (AJCC 7th Ed) [92]. 

Our group recently published the evolution of two cases of patients with metastatic NPC who, after progressing to treatment with immunotherapy with the anti-PD1 agent nivolumab, were rescued with platinum-gemcitabine, and where the dynamics of EBV-ctDNA in plasma correlated with the radiological response, demonstrating its role for the monitoring of advanced disease [93]. See Table 5.

### 2.3. Extracellular Vesicles

Extracellular vesicles (EVs) are non-nucleated lipid-layered round elements liberated from normal cells but also from tumor cells; they are implicated in multiple biological processes and harbor a rich protein and genetic (RNA and DNA) cargo, constituting an important reservoir of molecular information from their parental cells [9,10]. Tumor exosomes (TEX) may be isolated through different methods such as their biophysical properties or immune affinity, although ultracentrifugation is one the most used methods [4]. TEX are 30–150 nm EVs that are produced in high quantities by several solid tumors and are enriched in the plasma of patients with cancer, including SCCHN. Among their different roles, they perform major immunosuppressive functions in the tumor microenvironment [9]. Theodoraki et al. [94], in a study of 40 SCCHN patients, found that PD-L1^+^ TEX correlated with nodal and disease stage, while soluble PD-L1 in plasma did not. CD69 expression levels on T cells were inhibited by incubation with PD-L1^high^ TEX. Interestingly, in-vitro blockade of PD-L1^+^ TEX signaling to T cells diminished immune suppression. Recently, the same group, in a cohort of 17 SCCHN patients treated with surgery followed by adjuvant (chemo)radiation, demonstrated tumor exosomal protein decrease and tumor-/immune-cell derived exosomes decrease in responders, but an increase at the time of relapse, representing a promising liquid biopsy modality for early detection of relapse [95]. Among 18 SCCHN patients enrolled in a phase I trial and treated with cetuximab, ipilimumab and radiotherapy serial blood samples were collected for TEX and T cell-derived exosomes. While in patients who remained disease free, total exosome protein and TEX levels decreased, in patients with disease relapse, total exosomes protein and TEX levels increased [10]. Among 9 SCCHN treated with photodynamic therapy (PDT) blood samples were collected before and at 3 timepoints after therapy. TEX obtained before and 24 h after PDT were enriched in N-cadherin and TGF-β1 and enhanced tumor proliferation, migration and invasion. On the other hand, TEX from day 7 or from 4–6 weeks after PDT were enriched in E-cadherin and restored epithelial morphology and EpCAM expression in tumor cells, reduced the expression of mesenchymal genes and inhibited proliferation, migration and invasion, suggesting that PDT promotes the transition from a mesenchymal to an epithelial tumor phenotype mediated by TEX [96]. Wang et al. [97] compared the detection of miR-21 and HOTAIR in TEX between 52 patients with laryngeal SCCHN and 49 patients with polyps of the vocal cords. Levels of miR-21 and HOTAIR within TEX were significantly higher in patients with laryngeal carcinoma compared to patients with vocal cord polyps. Likewise, these miR-21 and HOTAIR levels within TEX were higher in laryngeal SCCHN patients with lymph node metastasis compared to those without.

### 2.4. MicroRNAs

MicroRNAs (miRNAs) are single-stranded non-coding RNAs of 18–25 nucleotides in length. They bind to the 3′-UTR of messenger RNAs, thereby regulating gene expression at the post-transcriptional level by means of RNA degradation and/or translational inhibition. They are highly stable at high temperatures or low pH and are involved in multiple biological processes during cancer development such as cell proliferation, invasion and metastasis. Previously, miRNAs have been detected in different body fluids, including serum, plasma and saliva in patients with SCCHN, and might be a promising liquid biopsy modality in this disease [97,98,99].

Interestingly, in a study evaluating the role of miR-196a and miR-196b in the early detection of oral cancer, levels of these markers were analyzed in 53 healthy controls, 16 patients with pre-malignant lesions and 90 patients with oral cancer. The miR-196a and miR-196b in plasma were significantly upregulated in patients with oral premalignant lesions and in patients with oral cancer compared to healthy controls. The combined determination of miR-196a and miR-196b showed very high sensitivity and specificity for the diagnosis of patients with oral premalignant lesions and oral cancer [100].

In another study, the levels of miR-31 were analyzed in saliva and plasma of 45 patients with oral carcinoma, 10 patients with oral verrucous leukoplakia and 24 healthy controls. Salivary miR-31 was increased in patients with oral carcinoma compared to controls, but there were no differences between patients with oral leukoplakia and healthy controls. In addition, miR-31 levels were higher in saliva than plasma, and salivary miR-31 levels were significantly reduced in patients with oral cancer after tumor resection [101].

Among 41 patients with head and neck cancer, 66% of whom had NPC and locally advanced disease, long non-coding RNAs (lncRNAs) lncRNA-p21, GAS5 and HOTAIR were analyzed in peripheral blood before and 4.5 months after therapy. Pretreatment GAS5 levels were significantly higher in patients with partial response/progressive disease compared with those achieving a complete response [102].

A meta-analysis identified miR-21 and miR-93 as the most upregulated miRNAs in SCCHN, while miR-9, miR-203, miR-375 are commonly downregulated [98].

A recent meta-analysis of 17 studies including 759 subjects demonstrated that miRNAs detected in saliva of patients with SCCHN had a 69.7% sensitivity and a 86.8% specificity, with an area under the curve (AUC) of 0.803 and a high diagnostic odds ratio (DOR) reaching 12.915 [99].

Finally, another meta-analysis of 25 studies including 2562 subjects with oral cavity cancer found a 78% and 82% diagnostic sensitivity and specificity, respectively, for blood and salivary miRNAs, with an AUC of 0.91 and a high DOR of 21.46 [103].

## 3. Future Perspective

The definitive implementation of liquid biopsy in HNC requires the clinician to have knowledge of two fundamental aspects: the molecular biology of cancer and the biological and biotechnological foundations for the use of each liquid biopsy modality. The future of liquid biopsy will be shaped by the joint use of different modalities and their combination with tumor imaging tests to provide more sensitive and refined information for clinical practice. It is probable that ctDNA will become the ideal tool in the short-midterm for the diagnosis, monitoring and measuring of MRD in viral-associated head and neck cancers such as HPV-related OPC and EBV-positive NPC. However, further prospective specifically designed studies are needed to demonstrate their role in treatment de-escalation strategies [65,67,70,78,94].

CTCs and EVs constitute some of the sources of molecular information with the greatest potential, as they allow multiple biological components to be analyzed, in contrast to ctDNA or miRNAs. However, important advances are still required to simplify their study and reduce costs [5]. The study of specific metabolites and metabolic signatures have shown interesting results in a few studies in patients with cancer, including SCCHN, and may demonstrate a promising role in a near future [4]. 

The role of saliva as the biological sample to be liquid biopsied is on the rise and will probably show promise as a screening strategy in premalignant or early malignant oral cavity and oropharyngeal cancers [6,104]. 

Finally, there is a potential role for liquid biopsy, probably in association with telemedicine, in early-disease diagnosis and monitoring, especially in resource-limited settings, since a well-organized and centralized web-like infrastructure for sample collection and rapid ctDNA analysis could serve as an initial screening allowing to tailor further and more complex investigations [105]. 

Several ongoing observational studies are evaluating the role of liquid biopsy in HNC and are summarized in Table 6.

## 4. Conclusions

The liquid biopsy field in head and neck cancer is a growing area of research that has demonstrated the feasibility of detecting CTC, ctDNA, EVs and miRNAs for prognostic and/or predictive purposes. In particular, the detection of ctDNA in virus-associated cancers such as HPV-related OPC and EBV-positive NPC has shown notable prognostic and predictive roles and should be incorporated into the design of future clinical trials. Multigene panel NGS-driven plasma ctDNA detection seems a promising diagnostic tool in HPV-unrelated SCCHN. The different liquid biopsy modalities should be combined with imaging tests in order to maximize their diagnostic accuracy for head and neck cancers.

## Figures and Tables

**Table 1:** Most relevant studies with CTC in patients with HPV-unrelated SCCHN.

Author	CTC Detection Technology	N	Stage	N (Samples)	Detection Rate	Prognostic Value
Wirtshafter (2002) [16]	CellSearch	18	I-IV	18	44% (0–3 CTC)	-
Partridge (2003) [17]	IA with negative enrichment	36	I-IV	36	50% (0–5 CTC)	Worse DFS
Guney (2007) [18]	CellSearch	21	LA	21	33%	-
Winter (2009) [19]	ISET (size)	16	LA	32 (pre- and post-SX)	63%	-
Jatana (2010) [20]	IA with negative enrichment	48	I-IV	61	71%	Worse DFS
Buglione (2012) [21]	CellSearch	73	I-IV	41 (pre- y post-TX)	15% (0–43 CTC)	Decrease in CTC → better respone
Nichols (2012) [22]	CellSearch	15	LA	15	40%	Worse OS in CTC+
Bozec (2013) [23]	CellSearch	49 (LA) 10 (HC)	LA	49 LA SCCHN 10 HC	16% SCCHN 0% in N(-) 23% in N1-2c 0% HC	-
He (2013) [24]	CellSearch	9	III-I	9	33% (0–1 CTC)	-
Hsieh (2015) [25]	IA with negative enrichment	53	LA or R/M	53	19%	-
Hristozova (2011) [26]	Fluid Cytometry	42	Unresectable LA	42	43%	Association of CTC+ with N+
Gröbe (2013) [34]	CellSearch	110	Resected (R0) OSCC	110	-	Association of CTC+ with N+ and <PFS
Tinhofer (2014) [27]	Immunoaffinity through tumor-antigen amplification	144	Resected	144	29%	Association of CTC+ with <DFS and OS
Grisanti (2014) [28]	CellSearch	53	R/M	53	26%	Association of CTC+ with <DFS and OS
Inhesten (2015) [29]	Fluid Cytometry	40	II-IV	120 (before, during and after TX)	97% (80% at baseline)	Association of CTC+ with <DFS and OS
Dyavanagoudar (2008) [37]	Detection of CK19 with RT-PCR	25	LA OSCC	25	16%	-
Kusukawa (2000) [30]	Detection of CK19 with RT-PCR	20	LA OSCC	20	10%	-
Garrel (2019) [31]	CellSearch, EPISPOT, Fluid Cytometry	65	R/M	Baseline, d7 and d21 after first cycle of EXTREME	EPISPOT: 69%, CellSearch: 21% CMF: 11%	Association of stability/increase of CTC with EPISPOT or CTC+ with CellSearch with <SLP
Strati (2017) [35]	CellSearch	113	LA	Baseline, after iCT and after CRT	PDL1 overexpression in CTC in 25.5% (baseline), 23.5% (after iCT) and 22.2% (after CRT)	Associaton of PDL1+ CTC post-CRT with <PFS and OS
Chikamatsu (2019) [32]	IA with negative enrichment and mRNA expression of epithelial markers (CK19, EpCAM, EGFR, c-MET)	30	R/M	30	CTC with epithelial marker expression ≥ 1:80%	-
Kulasinghe (2017) [33]	Spiral microfluidic system	1	R/M	1	Detection of CTC “clusters” with PDL1 expression	-
Kulasinghe (2016) [36]	IA vs. NIA: CellSearch (IA) vs. ScreenCell (NIA) vs. RosetteSep (NIA)	43	R/M	43	CellSearch: 18.6% (8/43) ScreenCell: 46.4% (13/28) RosetteSep 64% (16/25)	-
Liao (2019) [38]	IA with negative enrichment	20	LA or R/M	20	Detection of CTC with epithelial phenotype (E-CTC) in 75% and CTC with mesenchymal phenotype (M-CTC) in 90%	Association of M-CTC with higher odds of distant metastases and shorter PFS
Zheng (2019) [39]	CytoSorter (IA + microfluidic system)	20 (LA) 6 (LTB) 12 (HC)	LA	Pre- and post-iCT	HC: 0% CTC+ SCCHN: 77% CTC+	Association of CTC with N+, PR vs. CR and <DFS
Chang (2019) [40]	IA with negative enrichment	34	R/M	34	-	<DFS and OS and a higher CTC count
Wang (2019) [41]	IA with negative enrichment	53	LA	Before and during CRT	-	Association of CTC reduction with longer PFS and OS
Kawada (2017) [42]	CellSieve (Low pressure microfiltration system)	32	R/M	32	90.6%	Association of higher number of CTC with more advanced stage
Onidani (2019) [15]	LFIMA (microfluidic and inertial detection system) vs. ctDNA	9	R/M	9	Mutations in CTC in 4/9 pts and in ctDNA in 1/9 pts	-
Morgan (2019) [43]	SERS (RAMAN-type spectroscopy)	82	LA	82	-	Association of higher number of CTC with DMFS
Sun (2017) [44]	Meta-analysis of 17 CTC studies in SCCHN	-	-	-	-	Association of higher number of CTC with <PFS and <OS

SCCHN: head and neck squamous cell carcinoma, CR: complete response, CK19: cytokeratin 19, CO: oral cavity, CS: healthy controls, CTC: circulating tumor cells, ctDNA: circulating tumor DNA, DMFS: distant metastasis-free survival, DFS: disease-free survival, FCM: flow cytometry, HPV: human papillomavirus, IA: immunoaffinity detection method, ISET: isolation by size of epithelial tumor cells, LA: locally advanced disease, n: number, N: locoregional cervical nodes, NIA: non-immunoaffinity detection method, OS: overall survival, OSCC: oral squamous cell carcinoma, PFS: progression-free survival, PR: partial response, R0: complete tumor resection with free margins, R/M: recurrent and/or metastatic phase, RT-PCR: real-time PCR, SX: surgery, TBI: time to control of disease, TX: therapy.

**Table 2:** Most relevant studies with CTC in patients with EBV+ NPC.

Author	CTC Detection Technology	N (Patients)	Stage	N (Samples)	Detection Rate	Prognostic Value
Zhang (2018) [46]	SE-iFISH	50	II-IV	Pre- and post-CT	92%	Higher CTC count with a higher TNM/AJCC stage CTC count correlated with tumor response Cr 8 aneuploidy in CTC associated with CT efficacy
Zhang (2018) [46]	ISET	33	I-IV	33	66.7%	Good correlation of CTC count with EBV ctDNA
Si (2016) [47]	CanPatrol™ (size detection method) EBV-ctDNA detected with RT-PCR	81	I-IV	81	CTC detection: 96.6% 3 phenotypes: hybrid: >expression of EBV proteins, mesenquimal: >MMP-9 expression	Higher CTC count correlated with N+ and M1 disease
You (2019) [48]	CTC vs. EBV-ctDNA	148 R/M 122 LA	148 R/M 122 LA	Pre- and post-CT	M0: 19/122 (16%) M1: 64/148 (43.2%)	CTC and EBV-ctDNA have prognostic value that increases when combined between them and with tumor imaging tests
Li (2018) [49]	CTC and COX-2 expression in CTC	131	LA	Pre- and post-CRT	66.4% COX2+ CTC at baseline 46.1% COX2+ CTC post-CRT	< COX-2 expression post-CRT Worse prognosis with COX2+ CTC post-CRT
Vo (2016) [50]	CTC (Microsieve) vs. EBV-ctDNA (qPCR and dPCR)	46	LA	Pre- and post-CRT	EBV-ctDNA: qPCR BamHi-W 89%, qPCR EBNA1 69%, dPCR EBNA1 85%	Better correlation of clinical stage, radiological response and OS with EBV-ctDNA compared to CTC count
He (2017) [51]	ISET + IHQ CK5/CK6/P36 ISET + ISH EBERs	33	LA	Baseline	CTC: 66.7% CTM: 6.1%	Correlation of CTC count and titles of EBV VCA-IgA and EBV-ctDNA
Fu (2017) [52]	mRNA-hTERT in plasma and in CTC	33 NPC 24 HC	LA	Pre- and post-CRT	-	Association of mRNA-hTERT in plasma and CTC with clinical stage and response to therapy
Wen (2019) [55]	CanPatrol technology	60 NPC 18 HC	LA	Pre- and post-iCT	CTC+: 86.7% CTCmesenq+: 50%	Reduction of CTC count with iCT Worse prognosis with CTCmesenq+
Sun (2019) [54]	CellSpoter Analyzer vs. EBV-ctDNA (RT-PCR)	M0: 114 M1: 136	M0 & M1	Baseline	Median number of CTC: M0: 9.3 M1: 12.5	CTC count and LMP1, BART and EBER1 levels higher in M1 vs. M0
Ou (2019) [53]	CellSearch	370	III-IV	Baseline	M0: 77/288 (27%) M1: 16/81 (20%)	Worse prognosis with higher CTC count Combination of CTC and EBV-ctDNA have a better prognostic value
Xie (2019) [56]	CanPatrolTM + HIS (COX-2) vs. EBV-ctDNA (RT-qPCR)	50 II-IV 10 HC	II-IV	50 NPC 10 HC	CTC+: 96% M-CTC+: 76%	High CTC, M-CTC+ and COX2-CTC+ more frequent in stage IV and with EBV-ctDNA

AJCC: American Joint Committee on Cancer, CK: cytokeratin, CRT: chemoradiotherapy, CT: chemotherapy, Cr: chromosome, ctDNA: circulating tumor DNA, dPCR: digital PCR, mRNA: messenger RNA, CTC: circulating tumor cells, EBERs: EBER1 and EBER2 are EBV noncoding RNAs, EBV: Epstein-Barr virus, HC: healthy controls, iCT: induction chemotherapy, IHC: immunohistochemistry, ISET: Isolation by Size of Tumor Cells, L/LA: localized/locally advanced phase, MMP-9: matrix metalloproteinase 9, M0: non-metastatic stage, M1: metastatic stage, Mesenq: mesenchymal, NPC: nasopharyngeal cancer, n: number, N: locoregional cervical nodes, qPCR: quantitative PCR, RT-PCR: real-time PCR, SE-iFISH: subtraction enrichment and immunostaining-fluorescence in situ hybridization, TNM: tumor, node, metastasis classification.

**Table 3:** Most relevant studies with ctDNA in patients with HPV-unrelated SCCHN.

Author	N	Setting	Technology	Detection Rate	Specificity	Conclusions
Lin (2018) [64]	121 SCCHN (OC) 50 HC	LA	dPCR	-	-	ctDNA level higher in SCCHN vs. HC Higher levels of ctDNA with higher T stage and N stage Reduction in levels of ctDNA in 75% of cases after surgical resection
Van Ginkel (2017) [71]	6 SCCHN	LA	dPCR	100%	100%	Detection of mutations in 100% of cases
Egyud (2019) [72]	8 SCCHN	LA	NGS	6/8	-	Relapse in 4/8 pts, in 2 of them ctDNA detectable before relapse
Perdomo (2017) [65]	36 SCCHN plasma	LA	NGS (TP53, NOTCH1, CDKN2A, CASP8, PTEN)	18/36 (67%) (stages I and II)	-	Detection of 18 concordant mutations between primary tumor and plasma
Perdomo (2017) [65]	37 SCCHN plama & oral rinses	LA	NGS (TP53)	3/37	-	Low concordance of TP53 mutations between the primary tumor, plasma, and oral washings
Fostira (2019) [66]	54 LA SCCHN 15 R/M SCCHN	LA and R/M	SAFESEQ (TP53, CDKN2A, PIK3CA, HRAS)	LA: 51% R/M: 93%	-	- High tumor-plasma concordance, especially in R/M - Detection of emerging mutations during treatment or after PD
Flach (2022) [67]	17 LA SCCHN	LA	FFPE: WES Plasma: NGS deep sequencing (RaDaR^TM^)	Pre-SX: 100% Post-SX:	-	High tumor-plasma concordance in pre-SX samples All relapses showed post-SX ctDNA positive samples
Li (2020) [68]	247 R/M SCCHN	R/M (2nd line)	NGS Pre-TX	-	-	High bTMB (≥16 muts/Mb) predicted a longer OS and PFS with IO. Mutations in KMT2D or ATM in plasma ctDNA predicted longer OS with Durvalumab + Tremelimumab compared to CT
Porter (2020) [69]	60 R/M HNC (21 OPC, 8 SGC, 4 Thyroid cancer)	R/M	NGS (Guardant360)	SCCHN: 66% SGC: 50% Thyroid: 100%	-	Most common mutations in plasma: TP53 (68%), PIK3CA (34%), NOTCH1 (20%), ARID1A (15%). These results were concondant with tumor NGS, although 73% had blood alterations not identified in tissue. Alterations found in ctDNA allowed to inform management decisions.
Wilson (2021) [70]	75 R/M SCCHN	R/M	NGS	65% with actionable ctDNA alterations	-	Concordance among altered genes between tumor and ctDNA was 13% ctDNA alterations were significantly associated with decreased OS and presence and extent of disease at last visit.

CT: chemotherapy, ctDNA: circulating tumor DNA, dPCR: digital PCR, FFPE: formalin-fixed parafin-embeded, IO: immunotherapy, LA: locally advanced disease, HC: healthy controls, N: number, NGS: next generation sequencing, OC: oral cavity, OS: overall survival, PD: progression, PFS: progression-free survival, R/M: recurrent and/or metastatic phase, SCCHN: squamous cell carcinoma of the head and neck, SX: surgery, WES: whole exome sequencing.

**Table 4:** Most relevant studies with ctDNA in patients with HPV-related SCCHN.

Author (Year)	N	Setting	Technology	Detection Rate	Specificity	Conclusions
Damerla (2019) [75]	97 HPV(+) SCCHN (OPC) 6 HPV(−) SCCHN 20 HC	Localized	dPCR	95.6%	100%	ctDNA detected HPV16 and 33 with same accuracy that in tissue HPV-ctDNA also detectable in small tumors
Chera (2019) [76]	103 SCCHN	Localized	dPCR	89%	97%	None of the pts with ≥ 95% of ctDNA clearance relapsed 35% of the pts with <95% of ctDNA clearance relapsed HPV-ctDNA should be explored as a marker for deintesification strategies in HPV(+) disease
Mazurek (2016) [80]	200 SCCHN (HPV(+) and HPV(−))	Localized	RT-PCR (TERT amplification and HPV16/HPV18)	14% HPV+ in plasma	-	Higher HPV-ctDNA levels in OPC vs. other locations Higher HPV-ctDNA levels with higher stages Similar ctDNA levels in HPV(+) and HPV(−) SCCHN
Wang (2015) [6]	93 SCCHN	Localized	HPV(+): PCR digital (E6 y E7), PCR multiplex (E6 y E7) HPV(−): NGS (TP53,PIK3CA, CDKN2A,FBXW7, HRAS, NRAS)	L/LA: 10/10 (100%) R/M: 37/39 (95%) Saliva: OC (100%), Other (47–70%) Plasma: OC (80%), Other (86%)	-	High detection rate in plasma and saliva ctDNA detection in 3/3 cases that relapsed and in 0/5 cases that did not relapse
Dahlstrom (2015) [77]	262 SCCHN	Localized (I-IV)	RT-PCR	60.5%	67%	Baseline HPV-ctDNA associated with global and N stage Baseline HPV-ctDNA was not a predictor of relapse
Cao (2012) [78]	40 HPV(+) 24 HPV(−) 10 HC	Localized	RT-PCR	65%	-	HPV-ctDNA negativization after RT in 14 pts Increase in HPV-ctDNA in 3 pts with metastatic relapse
Ahn (2014) [79]	93 plasma and saliva pre- and post-TX (81 HPV(+) y 12 HPV(−))	Localized	RT-PCR	67%	89%	OS shorter in pts with detectable HPV-ctDNA post-TX in plasma or saliva
Siravegna (2021) [81]	61 HPV(+) SCCHN 45 HPV(−) SCCHN 25 HC	LA newly diagnosed SCCHN	ddPCR (HPV 16,18,33,35, 45)	98.4%	98.6%	Very high detection rates, with lower cost and earlier diagnosis compared to standard clinical workup
O’Boyle (2022) [82]	33	L/LA treated with surgery	ddPCR (HPV 16, 18, 33, 35, 45)	-	-	ctDNAHPV levels on POD 1 were associated with residual disease
Akashi (2022) [83]	25 HPV(+)	L/LA newly diagnosed SCCHN	dPCR (E6 & E7 regions of HPV DNA)	56%	-	56% detection rate at baseline. 0% detection rate after treatment. In 2 relapsing patients, HPV-specific ctDNA was positive.

ctDNA: circulating tumor DNA, dPCR: digital PCR, ddPCR: droplet digital PCR, HC: healthy controls, HPV: human papillomavirus, L: localized disease, LA: locally advanced, OC: oral cavity, OPC: oropharyngeal cancer, POD: postoperative day, RT: radiotherapy, RT-PCR: real-time PCR, SCCHN: head and neck squamous cell carcinoma, TX: therapy.

**Table 5:** Most relevant studies with ctDNA in patients with EBV+ NPC.

Author	N	Setting	Technology	Detection Rate	Conclusions
Chen (2018) [84]	385	Stage II NPC	RT-qPCR	161/385 (41.8%)	EBV-ctDNA levels and tumor volume allows to classify stage II NPC into favorable and unfavorable prognostic groups
Zhang (2015) [85]	1467	Stage I-IVB NPC	RT-qPCR	-	EBV-ctDNA levels complement TNM improving its prognostic value
Guo (2019) [86]	1529	Stage I-IVA NPC	RT-qPCR	-	EBV-ctDNA levels complement TNM improving its prognostic value
Lee (2019) [87]	518	Stage I-IVA NPC	RT-PCR	Median baseline EBV-ctDNA: 588 copies/mL	EBV-ctDNA levels complement TNM 8ª Ed improving its prognostic value
Liu (2015) [90]	185	Stage III-IVA NPC	RT-qPCR	Pre- iCT: 89% Post-iCT: 31%	Detectable EBV-ctDNA post-iCT associate with a worse Px
Xu (2019) [88]	2692	Stage III-IVA NPC	RT-qPCR	Pre- iCT EBV-ctDNA ≥ 2000 copies/mL: 57.5%	High levels of EBV-ctDNA pre-iCT associate with a worse Px and identify a group that benefits from iCT
Huang (2019) [89]	278	Stage III-IV NPC	RT-qPCR	Pre-iCT median EBV-ctDNA levels: 9035 copies/mL Post-iCT: 23.7%	High levels of EBV-ctDNA post-iCT associate with a worse Px
Zhang (2018) [91]	4482	Stage III-IVB NPC	RT-qPCR	Median EBV-ctDNA: 3740 copies/mL	High levels of EBV-ctDNA before iCT identified a poor-prognosis group that benefits from iCT
Chan (2017) [92]	20,349 screened → 309 ctDNA EBV+	Stage I-IVB NPC	RT-qPCR	Screening: 309/(5.5%)	Screening in an endemic population allowed to augment the % of detected cases in early stage (I–II) and this associated with a better survival.
Cabezas-Camarero (2020) [93]	2	Stage IV NPC	RT-qPCR	100%	Levels of EBV-ctDNA associated with response to CT post-IO

CT: chemotherapy, ctDNA: circulating tumor DNA, EBV: Epstein-Barr virus, HC: healthy controls, iCT: induction chemotherapy, IO: immunotherapy, LA: locally advanced disease, n: number, NGS: next generation sequencing, NPC: nasopharyngeal carcinoma, Px: prognosis, R/M: recurrent and/or metastatic phase, RT-PCR: Real-time PCR, RT-qPCR: Real-time quantitative PCR, TNM: tumor, node, metastasis classification.

**Table 6:** Current ongoing studies evaluating the role of liquid biopsy in head and neck cancer.

ClinicalTrials.gov (Accessed on 15 May 2022) (Other Study IDs)	Design	N	Sample Type	Primary Endpoint	Secondary Endpoint	Enrollment Status
NCT05122507 (KOHACIN study)	Prospective cohort study	200	Blood and saliva	Early recurrence detection lead time (time between liquid biopsy-based recurrence detection and clinical recurrence or progression)	RFS, OS	Recruiting
NCT03942380	Prospective cohort study	500	Blood (ctDNA, RNA, HPV-ctDNA)	% of HNC (all histologies) detected using liquid biopsy in blood % of HNC (all histologies) recurrence detected using liquid biopsy in blood	-	Recruiting
NCT03702309 (LIBERATE study)	Prospective cohort study	2500 (Several cancer types including HNC)	Archived tissue and peripheral blood	Collection and annotation of biospecimens at the Princess Margaret Cancer Center	Implement an electronic informed consent process for clinical research and correlative studies questionnaire at the Princess Margaret Cancer Center	Active, not recruiting
NCT04606940 (IO-KIN)	Prospective cohort study	20 (SCCHN)	Archived tissue and peripheral blood	Evaluate the kinetics of ctDNA in advanced/metastatic. SCCHN treated with anti-PD1 agents	- Changes in ctDNA levels in order to correlate with PFS and OS - Optimal time-point to analyze ctDNA as a predictive marker of response anti-PD1 agents	Recruiting
NCT04490564 (CBS-PD-L1a)	Prospective cohort study	25	Archived tissue and peripheral blood	Clinical performance of PD-L1 kit in CTCs of peripheral blood and tumor tissue samples.	Correlations between PD-L1 expression in serial liquid samples with patients’ responsiveness to therapy.	Recruiting
NCT05059444 (ORACLE)	Prospective cohort study	1000 (Several cancer types including HNC)	Archived tissue and peripheral blood	DRFi is defined as the time from the end of primary treatment until the time of diagnosis of a distant recurrence of the Index Cancer.	Sensitivity, PPV, Lead time defined as the interval between ctDNA detection and clinical detection of recurrence.	Recruiting
NCT04599309 (PRE-MERIDAN)	Prospective cohort study	20 (LA SCCHN candidates for standard definitive therapy)	Archived tissue and serially-collected peripheral blood	Number of high-risk LA-HNSCC patients with successful detection of ctDNA and/or HPV DNA in real time	- Correlation of presence of ctDNA +/− HPV DNA after standard treatment with shorter relapse-free survival (RFS), as assessed by comparison of baseline ctDNA +/− HPV DNA detection with time to relapse - Change in kinetics of ctDNA and/or HPV DNA over time after the end of standard definitive treatment and at recurrence, as assessed by ctDNA/HPV DNA analysis at sequential time points - Selection of the best time-point to detect MRD after standard definitive therapy in SCCHN, as assessed by comparison of quantified ctDNA +/− HPV DNA at 4–6 weeks vs. 8–10 weeks.	Recruiting
NCT03712566 (MASST-001)	Prospective cohort study	39 (Several cancer types including HNC)	Archived tissue and peripheral blood	To comprehensively characterize genomic, epigenetic and immune profiling features and changes in longitudinal blood samples that are associated with systemic treatment of recurrent or metastatic SCCHN.	- Establish a Clinically Annotated Biorepository - Correlate Multi-Omic Results with Clinical Outcome - Compare HPV-Positive and HPV-Negative Cell Histologies - Investigate the Relationship Between Genomic Profiles and Radiomic Signatures	Active, not recruiting
NCT05150509	Prospective cohort study	110 OSCC	Saliva	To establish a diagnostic test in the early detection of OSCC	-	Recruiting

ctDNA: circulating-tumor DNA, DRFi: distant recurrence-free interval, HNC: head and neck cancer, LA: locally advanced, HPV: human papillomavirus, OS: overall survival, OSCC: oral squamous cell carcinoma, PFS: progression-free survival, PPV: positive predictive value, RFS: relapse-free survival, SCCHN: squamous-cell cancer of the head and neck.
